# Improving extreme offshore wind speed prediction by using deconvolution

**DOI:** 10.1016/j.heliyon.2023.e13533

**Published:** 2023-02-06

**Authors:** Oleg Gaidai, Yihan Xing, Rajiv Balakrishna, Jingxiang Xu

**Affiliations:** a Shanghai Engineering Research Center of Hadal Science and Technology, College of Engineering Science and Technology, Shanghai Ocean University, China; b University of Stavanger, Norway

**Keywords:** Extreme wind speed estimation, Convolution, Reliability, Measured wind speed data, Offshore wind

## Abstract

This study proposes an innovative method for predicting extreme values in offshore engineering. This includes and is not limited to environmental loads due to offshore wind and waves and related structural reliability issues. Traditional extreme value predictions are frequently constructed using certain statistical distribution functional classes. The proposed method differs from this as it does not assume any extrapolation-specific functional class and is based on the data set's intrinsic qualities. To demonstrate the method's effectiveness, two wind speed data sets were analysed and the forecast accuracy of the suggested technique has been compared to the Naess-Gaidai extrapolation method. The original batch of data consisted of simulated wind speeds. The second data related to wind speed was recorded at an offshore Norwegian meteorological station.

## 1 Introduction and motivation

Over the years, there have been various strategies suggested for more accurate prediction of wind speeds. The countless strategies endeavoured included the method presented by Mohande [26], which predicts wind speeds using neural networks or the different approaches to analyse wind speed estimates by Yan et al., as in Refs. [[1], [2], [3], [4], [5], [6], [7], [8], [9], [10], [11], [12], [13], [14], [15], [16], [17], [18], [19], [20], [21], [22], [23], [24], [25], [26]]. Similarly, the authors of this paper have also previously used different statistical approaches to estimate better extreme statistical values such as, i.e. wind speed, wave height, response and load [[1], [2], [3], [4], [5], [6], [7], [8], [9], [10], [11], [12], [13], [14], [15], [16], [17], [18], [19], [20], [21], [22], [23], [24], [25], [26]]. Furthermore, the authors developed a novel reliability approach to forecast COVID-19 epidemic levels, indicating the approach's versatility and reliability in various fields [14,16,26]. Similarly, other authors, as in Refs. [[1], [2], [3], [4], [5], [6], [7], [8], [9], [10], [11], [12], [13], [14], [15], [16], [17], [18], [19], [20], [21], [22], [23], [24], [25], [26]], have attempted to use novel statistical approaches to estimate wind speed or pattern more accurately. Meanwhile, two recent and pertinent scientific articles [8,26] illustrated the importance of wind forecasting and strategies adopted to maximise efficiency. Like many of the papers mentioned above, the deconvolution methods implemented in this paper hope to accomplish better extreme offshore wind speed prediction.

With decades of studies in the field of extreme wind speed prediction in wind engineering, an accurate prediction of extreme values for engineering dependability tasks has become existential. It is significantly more critical whenever the data is insufficient. Thus, the need to develop new, reliable, efficient, and precise wind speed distribution tail extrapolation methods is of tremendous practical value. A dynamic statistical approach will give better estimates and results, which is the aim of the following approach.

Storms like tornadoes, hurricanes, gales, etc., create extreme wind speeds at turbine locations. These extreme wind speeds exert excessive loads on wind turbine parts, which can fail. In engineering design, such as a wind turbine, it is essential to obtain detailed information on the likelihood of extreme wind speeds. The suggested method is clear-cut and easy to understand when estimating extreme wind speed. It is envisaged that its application will be a valuable tool in future engineering design.

Consider a stationary stochastic process X(t) that is made up of the sum of two independent, component-level, stochastic processes, $X_{1}\left(t\right)$ and $X_{2}\left(t\right)\text{.}$

(1) $$X\left(t\right)=X_{1}\left(t\right)+X_{2}\left(t\right)$$

X(t) can be obtained either using simulations or measurements or a combination of both over a time period 0≤t≤T .

It is possible to derive the marginal probability density function (PDF) $p_{X}$ of X(t) in two ways. The first method is to measure directly $p_{X}^{A}$ from the available data measured in X(t); $p_{X}^{A}$ is the measured counterpart of the actual $p_{X}$. The second method is to obtain the component-level PDFs $p_{X_{1}}$ and $p_{X_{2}}$ independently from $X_{1}\left(t\right)$ and $X_{2}\left(t\right)\text{.}$ In this case, convolution can be applied on the measured counterpart $p_{X}^{B}=conv\left(p_{X_{1}},p_{X_{2}}\right)$ of the actual $p_{X}$. The actual $p_{X}$ can therefore be approximated by both $p_{X}^{A}$ and $p_{X}^{B}$.

It is intuitive to understand that it is much easier to obtain $p_{X}^{A}$ as it is a direct measurement from the data available in X(t). $p_{X}^{B}$, on the other hand, would require sufficiently accurately estimated $p_{X_{1}}$ and $p_{X_{2}}$ in order to perform an accurate convolution. However, that being said, even though the calculation process for $p_{X}^{A}$ is much more straightforward, a much longer time series is required to represent the extreme values. The extreme values are variables of low probability occurrence and do not appear in shorter time series. The convolution $p_{X}^{B}=conv\left(p_{X_{1}},p_{X_{2}}\right)$ provides for extrapolation and, therefore, could accurately represent the extreme values of the actual $p_{X}$. The convolution technique also does not assume any specific extrapolation functional class. This is in contrast to many traditional extrapolation methods widely used in engineering practice where an assumed probability function is used [[1], [2], [3], [4], [5], [6],^30^]. Some other examples also include the Pareto-based distribution peak over the threshold (POT) [1], The Naess-Gaidai (NG) method fitting procedure was used in averaged conditional exceedance rate (ACER) method [[7], [8], [9], [10], [11]], bivariate ACER fitting method [[12], [13], [14], [15], [16],^47^], Weibull distribution-based fitting method [38] and Gumbel distribution based fitting method [17].

The component-level PDFs $p_{X_{1}}$ and $p_{X_{2}}$ often not directly available, i.e., they are unlikely to be directly measurable. However, they can be artificially estimated using the following approach. In the simplest case, $X_{1}\left(t\right)$ and $X_{2}\left(t\right)$ can be defined to be identical stochastic processes that are equally spaced. This means $p_{X_{1}}=p_{X_{2}}$ and now the convolution can be written as

(2) $$p_{X}=conv\left(p_{X_{1}},p_{X_{1}}\right)$$

This is now a convolution of a single PDF and, therefore, only $p_{X_{1}}$ needs to be estimated.

The above is demonstrated using two example cases.

I) synthetic wind speed data – 100 independent and synthetically manufactured annual wind speed measurements
II) measured wind speed data – real wind speed measurements from Svenner Fyr

Case I) uses Monte Carlo simulations to synthetically manufacture annual peak events. Following [[27], [28], [29], [30], [31]], it is reasonable to assume the use of a stationary Gaussian process U(t) with mean = 0 and standard deviation = 1 to represent this case. Correspondingly, $\nu^{+}\left(0\right)T=10^{3}$ where $\nu^{+}\left(0\right)$ is the mean value up-crossing rate and T=1 year. Assuming that the up-crossing events are independent, the Poisson assumption can be used, and the following extreme value distribution over 3.65 days can be used to construct the synthetic data time series

(3) $$F^{3d}\left(\xi \right)=\exp\left\{-q\;\exp\left(-\frac{\xi^{2}}{2}\right)\right\}$$

where it was assumed $q=\nu^{+}\left(0\right)T^{3d}=10$. Eq. (3) therefore, represents the cumulative density function of a single wind speed maximum over a period of $T^{3d}=3.65$ days. This means an annual occurrence of 10^3^ mean zero up-crossings per year. Note that Eq. (3), i.e., case I) provides a method to generate synthetic data time series, having a purely illustrative purpose, and does not contain any particular engineering interpretation. The latter engineering interpretation will be addressed in case II).

**Fig. 1:**
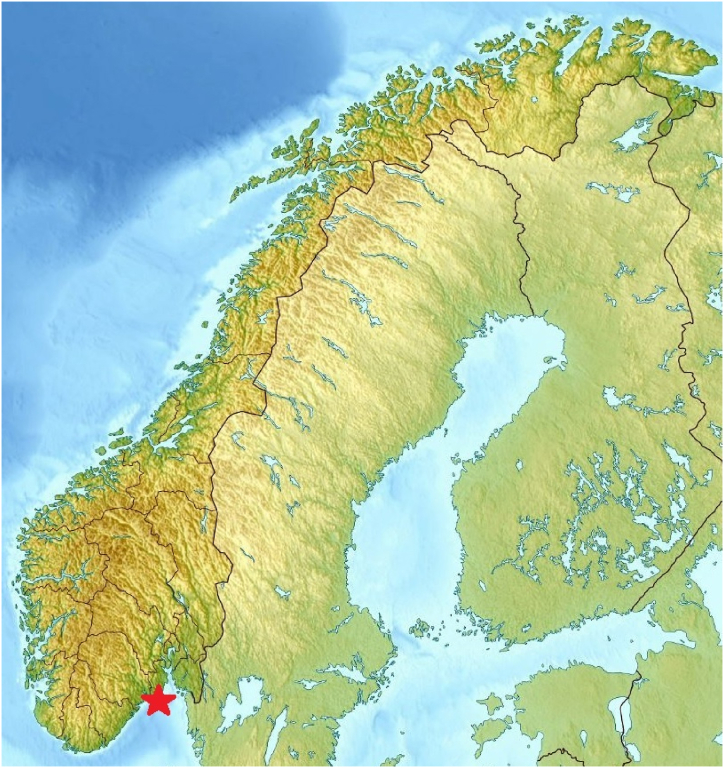
Map of Norway and neighbourhood with Svenner Fyr wind speed measurement station located offshore in Vestfold county, indicated by a star.

For case II) the authors utilised actual wind speed observations collected from Norwegian wind speed measurement station Svenner Fyr, located offshore in Vestfold county, Fig. 1. 6 h of wind speed maxima, recorded from 2008 to 2017, were downloaded from the Norwegian Meteorological Institute open-source, eKlima, [29]. Similar data can be obtained online for US offshore winds [46].

## 2 Discrete convolution

Convolution, w of two vectors, u and v can be represented using the following equation:

(4) $$w\left(k\right)=\sum_{j=1}^{m}u\left(j\right)v\left(k-j+1\right)$$

where j and k are vector indices, m=length(u) and n=length(v). This means that w has a total length of m+n−1. The summation is performed for all values of j in u(j) and v(k−j+1); specifically j=max(1,k+1−n):1:min⁡(k,m). Referring back to Eq. (4) where $p_{X_{1}}=p_{X_{2}}$, therefore, m=n since and therefore, this leads to Eq. (5)

w(1)=u(1)∙v(1)

w(2)=u(1)∙v(2)+u(2)∙v(1)

(5) w(3)=u(1)∙v(3)+u(2)∙v(2)+u(3)∙v(1)

w(n)=u(1)∙v(n)+u(2)∙v(n−1)+…+u(n)∙v(1)

w(2n−1)=u(n)∙v(n)

Having discovered u=v=(u(1),..,u(n)), in Eq (5), one may progressively derive the w-components w(n+1),..,w(2n−1), as the index grows from n+1 to 2n−1. The latter would extend vector w into double the length of the support domain of the original distribution, doubling the $p_{X}$ distribution support length $\left(2n-1\right)∙\Delta x\approx 2n∙\Delta x=2X_{L}$ relative to the original distribution support length $n∙\Delta x=X_{L}$ where Δx is the constant length of each discrete bin of the distribution. The convolution procedure extrapolates the distribution tail properties, i.e., the distribution tail is fitted and extended towards the direction of the very low probabilities of exceedance values. The values of u and v are defined to be zero outside of the vector. The last statement is false. See Section 3 for the linear extrapolation of vectors u and v on the logarithmic scale; u and v will be represented by the probability distribution function, $f_{X_{1}}$, and w will be represented by, $f_{X}$. w=(w(1),..,w(n)) is a discrete representation of the target empirical distribution. $p_{X}$ from Section 1, and n are representing the length of distribution support, $\left[0,X_{L}\right]$. Therefore, for simplicity in this paper, a one-sided distribution with only positive valued random variables is assumed, i.e., X≥0. This also suits well for wind speeds which are only positive. Further in accordance with Eq. (5), u=v. Therefore in accordance with Eq. (2), $p_{X}$ and $p_{X_{1}}$ are the corresponding estimated PDFs for w and u**,** respectively.

Given w=(w(1),..,w(n)) it is evident that one may successively discover the unknown components w=(w(1),..,w(n)) beginning with the first component $u\left(1\right)=\sqrt{w\left(1\right)}$, then the second $u\left(2\right)=\frac{w\left(2\right)}{2u\left(1\right)}$, and so on until u(n). The extrapolation scheme proposed by the authors is one that linear, i.e., the simplest form of extrapolation. This means (u(1),..,u(n)) will be deconvoluted with (u(n+1),..,u(2n−1)), i.e., linear extrapolation to the range of $\left(X_{L},2X_{L}\right)$ will be performed on the tail of PDF $p_{X_{1}}$. It is highlighted that $p_{X_{1}}$ is the deconvoluted PDF that estimates u**.** Subsequently, w is extended and extrapolated into twice the length of $p_{X}$, i.e., $\left(2n-1\right)∙\Delta x\approx 2n∙\Delta x=2X_{L}$, where $n∙\Delta x=X_{L}$ is the original length of $p_{X}$.

The interpolation of the distribution $p_{X}\left(x\right)\equiv p_{X}$ tail was carried out because the CDF's tail is often highly regular for large values of x. Owning to this regularity, the Naess-Gaidai (NG) approach can be used for $x\geq x_{0}$, where the tail is found to behave close to $\exp\left\{-{\left(ax+b\right)}^{c}+d\right\}$ with a,b,c,d being constants appropriate for $x_{0}$, see Eqs. (7), (8); for further information, see Refs. [[32], [33], [34], [35], [36], [37]]. The authors have considered linear extrapolation of $p_{X_{1}}$ tail as the simplest unbiased option. That being said, other non-linear extrapolation methods may also be used, but their appropriateness in relation to their inherent biases and assumptions must be considered when evaluating the accuracy [7].

## 3 Numerical results

This section presents the numerical results from two examples. The first example is a synthetic wind example, while the second is an actual field measurement case of wind speeds recorded at Svenner Fyr, a lighthouse in Southern Norway. The rationale for choosing synthetic wind is that the actual analytical solution and the actual extreme value statistics are known analytically. This would therefore allow for accurate validation of the proposed method.

The probability of exceedance (1-CDF) is assigned the notation $f_{X}$ in this paper. 1-CDF is also referred to in the literature as the complementary CDF (CCDF), and its estimation is essential in engineering reliability assessments. It is worthwhile to mention that $f_{X}$ is also similar to the marginal probability density function $p_{X}$ discussed in Section 1. Further, the proposed method can deal with both concave and convex CCDF function tails as long as they are sufficiently regular and monotonous decreasing.

The validation procedure is as follows. First, a «shorter » data subset is selected from the complete « longer » data set. Second, predictions are performed using the proposed method on the «shorter » dataset. Lastly, the predicted values are then compared and validated against the «longer » data set. In this paper, the «shorter » wind speed time series was set to be 10 to 100 times shorter than the original « longer » time series. In doing so, the paper would have proven that the proposed method has an efficiency of at least two orders of magnitude. The previously-discussed phenomenon of distribution tail almost linear dependence is confirmed in the case of the synthetic wind speed distribution given by Eq. (3), namely $F=\exp\left\{-q\;\exp\left(-\frac{\xi^{2}}{2}\right)\right\}\Rightarrow \ln\left(1-CDF\right)\approx \ln\;q-\frac{\xi^{2}}{2},\ln\;f={\ln\;F}^{\prime }\approx \ln\;q-\frac{\xi^{2}}{2}+\ln\;\xi$, and it is clear that lnξ varies much more slowly in the tail (i.e. for larger ξ) than a parabolic term $-\frac{\xi^{2}}{2}$. Due to the marginal PDF tail irregularity, there is a clear advantage of extrapolating 1−CDF instead of the marginal PDF. It is possible to perform this extrapolation using an iterative scheme like in the NG method [41]. Alternatively, it is also possible to use integration, e.g., deconvolution, to generate an artificial smoother CDF. This latter approach makes extrapolation easier in the case where the distribution is reasonably irregular due to insufficient data at the lower probabilities of exceedances.

The objective of solving of Eq. (5), i.e., the discrete convolution procedure, is to find $f_{X_{1}}$ given $f_{X}$, i.e., the deconvoluted 1 − CDF and the actual 1-CDF, respectively. Note that u=(u(1),..,u(n)) is normally monotonously decreasing and is the same for $f_{X}$. This means (u(n−L),..,u(n)) for some L<n may become negative. This is obviously a numerical error since there are only positive values in probabilities, and a scaling procedure as described has been introduced to mitigate this.

The pivot value is defined as the minimum positive value $f_{L}$ of $f_{X}$’s distribution tail. The scaling is performed in the y-direction on the decimal logarithmic scale in accordance with Eq. (6)

(6) $$g_{X}=\mu \left(\log_{10}\left(f_{X}\right)-\log_{10}\left(f_{L}\right)\right)+\log_{10}\left(f_{L}\right)$$

with $g_{X}\left(x\right)$ being the scaled $\log_{10}$ version of the empirical base distribution $f_{X}$, with the reference level $f_{L}$ being intact. The scaling coefficient μ=1/3 is chosen in this paper for both examples presented to avoid negative values in $f_{X_{1}}$. After estimating $f_{X_{1}}$, convolution is performed by calculating ${\tilde{f}}_{X}=conv\left(f_{X_{1}},f_{X_{1}}\right)$ according to Eq. (2). Note that ${\tilde{f}}_{X}$ is the extrapolated counterpart of $f_{X}$, see Eq (6). Further, inverse scaling to the original scale is performed with $\mu^{-1}$.

Finally, interpolation was necessary on the «shorter» $f_{X}$ because the empirical $f_{X}$ distribution is naturally highly irregular at the terminal tail section, thus making the empyrical $f_{X}$ distribution unsuitable input for Eq (5). The NG (Naess-Gaidai) method is used for the interpolation

(7) $$f_{X}\left(x\right)\approx \exp\left\{-{\left(ax+b\right)}^{c}+d\right\},x\geq x_{0}$$

where a,b,c,d are variables to be minimised with respect to the mean square error.

The integral form of Eq (7) is used for the extrapolation in this paper

(8) $$F\left(a,b,c,d\right)={\int_{x_{0}}^{X_{L}}w\left(x\right)\left\{\ln\left(f_{X}\left(x\right)\right)-d+{\left(ax+b\right)}^{c}\right\}}^{2}dx,x\geq x_{0}$$

where $x_{0}$ is the tail marker, defining the start of the extrapolation tail area. This is shown by the green squares in the right diagram of Fig. 3. It is mentioned that a,b,c,d in Eq. (8) are optimised using the Levenberg-Marquardt non-linear least squares algorithm [[43], [44], [45]]. Further, there exist alternative regression models [2] which also can be used.

### 3.1 Synthetic wind speed data

The 3.65-day maximum speed wind X(t) is considered in this example case. X(t) is generated for [0,T] where T=1 year. As mentioned previously in Section 1, the underlying process U(t) is assumed to be normal Gaussian with mean = 0 and standard deviation = 1. Therefore, the mean zero up-crossing rates of U(t) will satisfy $\nu_{U}^{+}\left(0\right)=10^{3}/T$ which is a common assumption used for offshore wind problems, [4]. The data set is divided into a «shorter» and a «longer » data record. The « shorter » record has $10^{4}$ data points (100 years) while the «longer » record is the complete data set and has $10^{6}$ data points (10,000 years). This means the data set for X(t) has 365/3.65 = $10^{2}$ data points (1 year).

The normal Gaussian U(t) then results in $F_{X}^{3d}\left(x\right)=\exp\left\{-q\;\exp\left(-\frac{x^{2}}{2}\right)\right\}$, where q=10. $F_{X}^{3d}\left(x\right)$ is the analytical expression for the CDF of the 3.65-day maximum wind speed X(t). Correspondingly, the non-dimensional X value for the 100-year 3.65-day maximum wind speed is $x^{100\;yr}=4.80$. An artificial horizontal axis shift $x\to x-x_{shift}$ with $x_{shift}=1.5$ is used ensure operating within the distribution tail. Further $x_{shift}=x_{0}$ in Eq. (7), i.e., $x_{shift}=1.5$ is selected as the cut-on tail market. Correspondingly, $x^{100\;yr}=4.80-x_{shift}=3.3$.

**Fig. 2:**
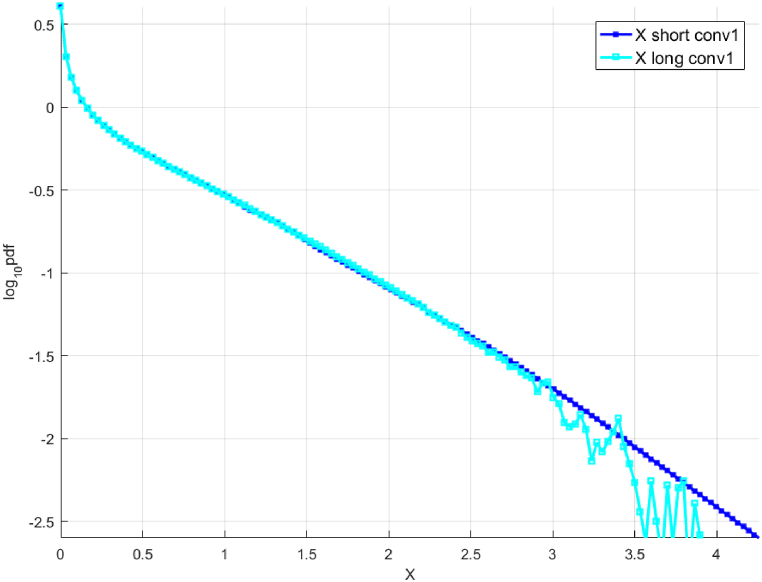
Synthetic wind speed data. Scaled $f_{X_{1}}$ tail for the «shorter » data (cyan squares) and «longer » data (blue squares). Presented in decimal log scale.

**Fig. 3:**
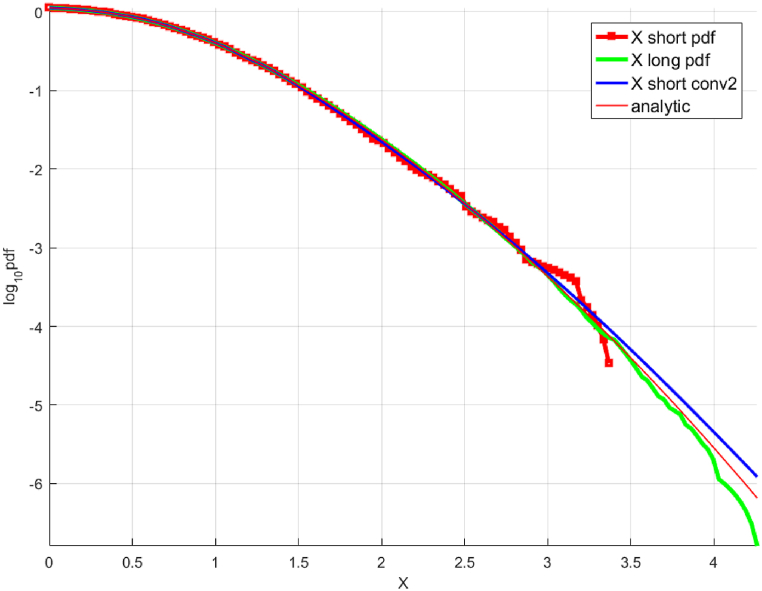
Unscaled « shorter» $f_{X}$ tail, raw (red squares) and fitted (solid blue line, along with «longer » data (green line) and analytic (solid red line). Presented in decimal log scale.

Fig. 2 presents the «shorter» and «longer » data sets. The « shorter » data set is scaled to present the results on the same horizontal scale as the «longer » data set. Fig. 3 presents the predicted distribution obtained by deconvolution extrapolation, the original analytical distribution, and the «shorter» (unscaled) and «longer » data sets. To recap, the «shorter» and «longer » data sets were generated using Monte Carlo simulations based on the exact analytical distribution. The « shorter » data set is also 100 times shorter than the «longer » one. Further, the NG extrapolation method [13] was used for additional comparison and validation. The results show that the 10,000-year 3.65-day maximum wind speed predicted by deconvolution is within 5% of the value predicted by the NG method and the actual value calculated using the analytical distribution.

As already mentioned, the advantage of the deconvolution extrapolation technique is that it is not confined to the pre-chosen decimal logarithmic scale form given by Eq. (7). Therefore, it offers more potential flexibility and accuracy in more challenging applications.

### 3.2 Measured wind speed data, Norway

**Fig. 4:**
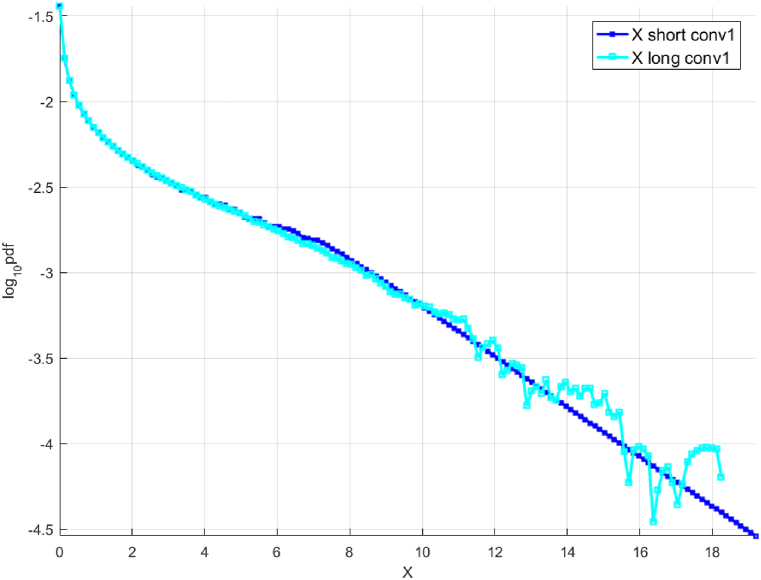
Measured wind speed data from the wind station Svenner Fyr. Scaled $f_{X_{1}}$ tail for the «shorter » data (cyan squares) and longer data (blue squares). Presented in the decimal log scale.

The Svenner Fyr wind station is located offshore in the Norwegian Vestfold county where 6-h intervals of wind speed maxima were measured for a period of 10 years from 2008 to 2017 were used. The « shorter » data set is created by selecting 1 in every 10 data point, i.e., only 1 year long of wind speeds, while the «longer » data set contains the full 10 years of wind speeds. Fig. 4 presents the scaled results. The method is applied to proper time duration stationarity windows, with the mean subtracted. According to the scattered diagram, seasonal wind speed variations are averaged – a standard engineering procedure linking short-term to long-term analysis.

**Fig. 5:**
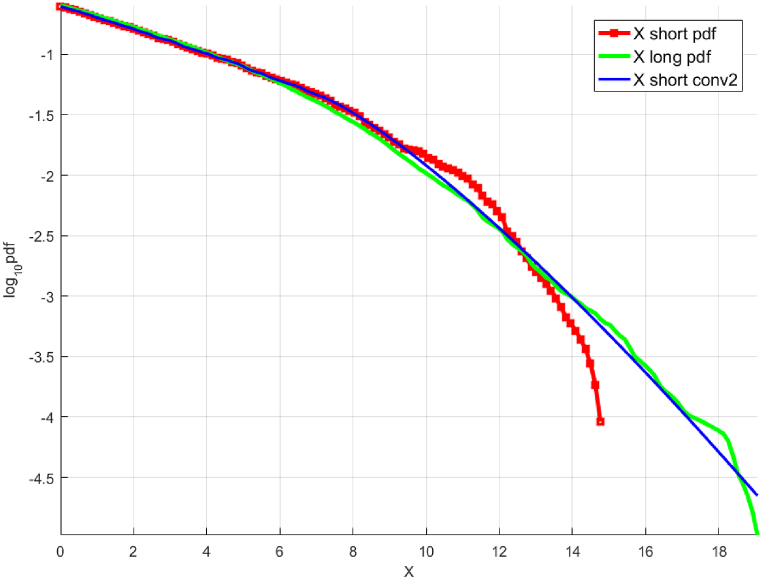
Unscaled « shorter » decimal log scale $f_{X}$ tail, raw (red squares) and extrapolated by deconvolution (solid blue line, along with «longer » data (green line). The horizontal axis is in m/sec.

Similar to Fig. 2, Fig. 3, Fig. 4, Fig. 5 present (i) «shorter» (scaled) and «longer » data sets and (ii) the predicted distribution obtained from extrapolation and the «shorter» (unscaled) and «longer » data sets respectively, when applied to the actual wind speeds measured at Svenner Fyr. As observed in Fig. 4, Fig. 5, both deconvolution extrapolation approaches lead to predictions that are reasonably similar to the «longer » data set. The authors acknowledge that only a single measured wind speed data set is used in this paper, and more validation studies are required to conclude the accuracy of the method proposed here robustly.

### 3.3 Measured wind speed data, Hawaii

An additional real-world example is presented to illustrate the proposed deconvolution method further. This example is the 10-min average wind speeds measured at Station 51,101 (LLNR 28006.3) in northwestern Hawaii, operated by National Oceanic and Atmospheric Administration (NOAA).

**Fig. 6:**
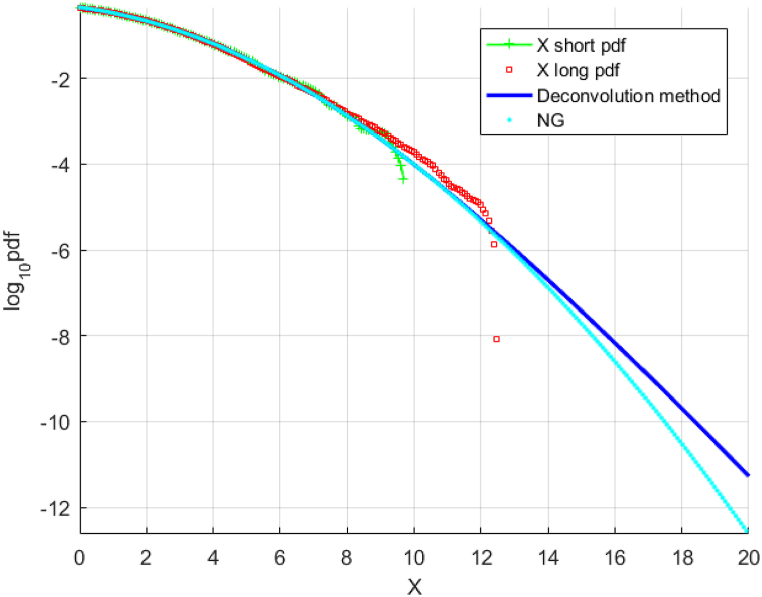
Wind speed data at Station 51,101, northwestern Hawaii. Unscaled « shorter » raw data (green) $f_{X}$ tail on the decimal log scale extrapolated using the deconvolution technique (dark blue), as well as scaled « longer » raw data (red) and NG extrapolation (cyan). A star denotes a return time of 100 years.

Fig. 6 exhibits good agreement between both compared methods. However, NG method yields slightly less conservative results than the suggested deconvolution method.

## 4 Conclusions

The major benefit of the deconvolution method proposed in this paper, which in contrast to most other traditional extrapolation, utilises the inherent statistical properties of the data set and does not utilise any assumed distribution functional class. This paper examines two sets of wind speed data, a synthetic and a measured set, to demonstrate the method’s accuracy and efficiency. The prediction accuracy is benchmarked against the NG method. For the case of synthetic wind speed, the proposed approach produced forecasts that agreed with both the analytical solution and the NG method. For the case of the actual wind speed measurements at Norwegian and Hawaii measurement stations, the deconvolution method can produce a distribution close to the complete ten-year data set by extrapolating the one-year data set. Further, the predictions provided by deconvolution were also consistent with the predictions obtained via the Naess-Gaidai method. Being an unbiased extrapolation method, the deconvolution method can be utilised specifically in instances where an unbiased characteristic design value is highly sought after. Lastly, the proposed deconvolution method is general and can be applied to a broad range of possible technical applications.

Ethics approval and consent to participate – conformed.

Consent for publication – obtained.

## Declarations

### Author contribution statement

Oleg Gaidai: Conceived and designed the analysis; Wrote the paper.

Yihan Xing: Analysed and interpreted the data; Wrote the paper.

Rajiv Balakrishna: Analysed and interpreted the data; Wrote the paper.

Jingxiang Xu: Contributed reagents, materials, analysis tools or data.

### Funding statement

This work was supported by the Shanghai Engineering Research Center of Marine Renewable Energy [grant number: 19DZ2254800].

### Data availability statement

Data will be made available on request.

### Declaration of interest’s statement

The authors declare no conflict of interest.

## Software and data availability

⁃ Authors used Matlab commercial software tools; see http://www.mathworks.com/
⁃ For ACER routines, see https://folk.ntnu.no/arvidn/ACER/
⁃ For extrapolation routines, see https://github.com/gocrane/crane
